# Effects of eplerenone on blood pressure and glucose metabolism in Japanese hypertensives with overweight or obesity

**DOI:** 10.1097/MD.0000000000014994

**Published:** 2019-04-12

**Authors:** Hisashi Adachi, Tatsuyuki Kakuma, Machiko Kawaguchi, Eita Kumagai, Yoshihiro Fukumoto

**Affiliations:** a Department of Community Medicine, Kurume University School of Medicine; b Biostatistics Center, School of Medicine, Kurume University; c Academic Research Organization, Kurume University; d Division of Cardio-Vascular Medicine, Department of Internal Medicine, Kurume University School of Medicine, Kurume, Japan.

**Keywords:** eplerenone, glucose metabolism, hypertension, obesity, trichlormethiazide

## Abstract

**Objective:**

The impact of aldosterone blockade using eplerenone on hypertensives with obesity has not been clarified. We compared the efficacy and safety between eplerenone and trichlormethiazide in hypertensives with overweight or obesity.

**Methods:**

A prospective, randomized, open-labeled, blinded-endpoint design, multicenter trial enrolled 204 hypertension-treated outpatients with obesity [body mass index (BMI) ≥25 kg/m^2^] evaluated by ambulatory blood pressure (BP) measurement. Patients were randomly assigned to receive 50 mg of eplerenone (*n* = 102) or 1 mg of trichlormethiazide (*n* = 102), each of which were administered once every morning. Primary efficacy endpoints were systolic and diastolic BPs and biomarkers of glucose metabolism after 6 months of treatment.

**Results:**

At baseline, BPs were comparable between the two groups. Systolic/diastolic blood pressure (SBP/DBP) were reduced from 153.9 ± 12.6/84.6 ± 11.8 to 129.8 ± 14.2/73.7 ± 12.2 mm Hg by eplerenone therapy and from 152.2 ± 12.5/85.2 ± 10.9 to 133.8 ± 12.6/76.1 ± 8.6 mm Hg by trichlormethiazide therapy (all; *P* < .001). The efficacy of SBP reduction after adjustment for age, sex, and BMI was significantly greater in the eplerenone group than the trichlormethiazide (*P* = .034), although the efficacy of DBP reduction was marginally significant (*P* = .072). Especially, the efficacy of BP reduction was more effective for aged over 65 years than less than 65 years. However, biomarkers of glucose metabolism were not significantly different between these 2 groups.

**Conclusion:**

The eplerenone therapy was more effective in BP lowering in hypertensives with overweight or obesity than the trichlormethiazide therapy, especially in the elderly.

## Abstract 2

Supplemental Digital Content is available in the text

## 1 Introduction

Hypertension with obesity is prevalent in Japanese population, which is crucial health problems since both hypertension and obesity are risk factors for cardiovascular events.^[1,2]^ A current guideline for hypertension (JSH2014) recommends prescribing angiotensin-converting enzyme (ACE)-inhibitors or angiotensin receptor blockers (ARBs), followed by thiazide diuretics.^[3]^ However, it is well known that thiazide diuretics have unfavorable effects in glucose metabolism.

Aldosterone plays a central role in hypertension, and hypertension is prevalent in patients with insulin resistance. We have previously investigated the significant relationship between plasma aldosterone levels and insulin resistance in 564 nondiabetic participants.^[4]^ The 10-year prospective study demonstrated that plasma aldosterone levels predicted the development of insulin resistance in a general population.^[4]^

Based on the findings from the prospective study, we hypothesized that the aldosterone blockade, eplerenone, is effective in hypertensives with obesity for lowering blood pressure (BP) as well as improving glucose metabolism compared to thiazide diuretics.

## 2 Methods

### 2.1 Study subjects

We enrolled Japanese obese patients with BMI ≥25 kg/m^2^, currently on antihypertensive medication, and assessed the effects of eplerenone versus trichlormethiazide on biomarkers of blood pressure, heart rate, and glucose metabolism over 6 months. The present study was approved by the Ethics Committee of Kurume University School of Medicine (protocol No. 12138) and registered in the University Hospital Medical Information Network (UMIN Clinical Trials Registry 000009127).

1. Registration criteria

(1) Inclusion criteria as follows: 1) Patients with SBP ≥140 mm Hg and/or DBP ≥90 mm Hg despite on antihypertensives; 2) Patients with BMI ≥25 kg/m^2^; 3) Patients treated with one or more antihypertensives (ARB, ACE-inhibitor, direct renin inhibitor (DRI), calcium channel blocker (CCB), alpha-blocker, or beta-blocker),

1. If patients were on drug for diabetes, the types and doses of the drugs were stable during 3 months prior to enrollment
2. Patients who provided written informed consents
3. Japanese males and females who were more than or equal to 20 years old at the time of giving informed consent

(2) Exclusion criteria as follows: 1) Patients on eplerenone or spironolactone during 3 months prior to enrollment; 2) Patients on diuretics including trichlormethiazide or combination drugs during 3 months prior to enrollment; 3) Patients who experienced myocardial infarction or stroke during 30 days prior to enrollment; 4) Patients who had cardiac surgery or percutaneous coronary intervention (PCI) during 30 days prior to enrollment; 5) Patients with serum potassium ≥5.0 mEq/L; 6) Patients with severe liver dysfunction; 7) Patients with moderate or severe renal dysfunction (Ccr < 50 ml/min); 8) Patients with grade III hypertension (SBP ≥180 mm Hg and/or DBP ≥110mm Hg); 9) Patients diagnosed as secondary hypertension; 10) Patients with hypersensitivity with eplerenone or sulfonamide derivative; 11) Patients who were contraindicated for use in eplerenone or trichlormethiazide; 12) Patients who were on medications contraindicated for use with eplerenone or trichlormethiazide; 13) Women who were either pregnant, lactating or of childbearing potential; 14) Patients who were considered not appropriate to enroll into this trial by the investigator.

2. Study treatments

(1) Screening

A. Pre-screening

1) Measure height and weight to calculate BMI
2) Measure SBP and DBP at office with seated position

B. Main-Screening

Conduct screening with following items on the patients who were BMI ≥ 25 kg/m^2^ and SBP ≥ 140 mm Hg and/or DBP ≥ 90 mm Hg based on the pre-screening measurement.

(1) Obtain informed consent
(2) Patient background:a. Age, b. sex, c. history of smoking, d. previous medical history (including duration of hypertension), e. other appreciable medical conditions
(3) Complications
(4) Concomitant medicationsa. ARBs and/or ACE-Is, b. CCBs, c. beta-blockers, d. diuretics, e. DRIs, f. Other concomitant medications (including drugs other than antihypertensives)
(5) Concurrent therapy
(6) Physical findings (including abdominal circumferences)
(7) Clinical laboratory tests [including serum potassium, serum creatinine, serum natrium, Ccr, estimate glomerular filtration rate (eGFR), aspartate aminotransferase (AST), alanine aminotransferase (ALT)]

C. After assignment of 2 groups

1. Eplerenone group

1) Keep the baseline medication(s) and administer eplerenone 50 mg/day.
2) If SBP was not controlled under 140 mm Hg or DBP was not controlled under 90 mm Hg, the dose of eplerenone was to be titrated up to 100 mg/day with careful monitoring for serum potassium level.
3) If necessary, antihypertensive medications such as ARB/ACE-I, DRI, CCB, alpha/beta-blockers could be added after 3 months of randomization.
4) Diuretics were not allowed to prescribe in this group.
5) If patients were prescribed cytochrome (CYP) 3A4 inhibitors (clarithromycin or verapamil hydrochloride, etc.), dosage of eplerenone should be kept as 25 mg/day and should not exceed the amount.
6) If serum potassium level increased to 5.5 mEq/L, patients should stop eplerenone. Restart taking eplerenone with lower dose only if serum potassium level dropped to less than 5.0 mEq/L.
7) As a general rule, the amount of concomitant medications should not be added, increased, or decreased during the study period, unless the investigators needed the modification.

2. Trichlormethiazide group

1) Keep the baseline medication(s) and administer trichlormethiazide 1 mg/day.
2) If SBP was not controlled under 140 mm Hg or DBP not controlled under 90 mm Hg, the dose of trichlormethiazide could be titrated up to 2 mg/day.
3) If necessary, antihypertensive medications such as ARB/ACE-I, DRI, CCB, alpha/beta-blockers could be added after 3 months of randomization.
4) Eplerenone and/or spironolactone were not allowed to prescribe in this group.
5) As a general rule, the amount of concomitant medications should not be added, increased, or decreased during the study period, unless the investigators needed the modification.

3. Concomitant medications and concurrent therapies

1) All medications and therapies used during the study period were considered as concomitant medications and concurrent therapies. All such practices were documented.
2) Following medications are not allowed to use during the study period.
  - Spironolactone
  - Potassium canrenoate
  - Potent CYP3A4 inhibitors and/or CYP3A4 revulsant (see also “4.2 Exclusion criteria”.)

D. Adverse event reporting

When adverse events were observed in patients participated in this study, the investigators reported them to the data center (Academic Research Organization, Kurume University) in accordance with the rules for reporting adverse events in usual clinical practice of the respective institutions.

E. Study period

Patient entry period: November 1, 2012 to March 31, 2016 (including screening period); Protocol amended on September 11, 2013.

Follow-up period: 6 months after enrollment (- September 30, 2016)

F. Endpoints

1) Primary endpoints:

- Change from baseline in office SBP/DBP after 24 weeks
- %Change from baseline in insulin sensitivity (homeostasis model assessment insulin resistance (HOMA-IR)) after 24 weeks
- %Change from baseline in hemoglobin A_1c_ (HbA_1c_) after 24 weeks

2) Secondary endpoints:

Changes from baseline in high sensitivity C-reactive protein (hsCRP), eGFR, adiponectin, and the use of potassium supplement

1. Safety parameters:
  (1) Serum potassium for up to 24 weeks
  (2) Adverse events, laboratory values, physical examinations, and vital signs for up to 24 weeks
2. Observation parameters
  (1) Patient background: Age, sex, height, weight, BMI, abdominal circumference, history of present illness, complications, past medical history, therapeutic situation, pretreatment
  (2) Office blood pressure/pulse: Systolic and diastolic blood pressure (sitting), heart rate
  (3) Blood chemistry: complete blood count, blood urea nitrogen (BUN), creatinine (Cr), electrolytes (Na, K, Cl), AST, ALT, gamma-glutamyl transpeptidase (γ-GTP), alkaline phosphatase (ALP), total bilirubin, total cholesterol, triglycerides, low-density lipoprotein (LDL) cholesterol, high-density lipoprotein (HDL) cholesterol, remnant-like lipoprotein cholesterol (RLP-c), fasting plasma glucose, HbA_1c_, insulin, adiponectin, cortisol
  (4) Insulin sensitivity: homeostasis model assessment insulin resistance (HOMA-IR)
  (5) Inflammatory markers: hsCRP
  (6) Renal function: eGFR (IDMS-MDRD equation method)
  (7) Renin-angiotensin system (RAS) components: (renin, angiotensin II, aldosterone)
  (8) Electrocardiography (ECG) (on a voluntary basis)
  (9) Compliance status: Calculation is performed by considering cases where all prescribed doses are taken as 100%.
  (10) Safety: Serum potassium, serum Na+, hepatic function test

## 3 Statistical analysis

Prospective, Randomized, Open, Blinded-Endpoint (PROBE)^[5]^ design was used.

The registration process was as follows:

1. Investigators confirmed that screened patients met the inclusion criteria and did not meet the exclusion criteria.
2. Investigators informed to a preassigned investigational staff member about patients who met registration criteria.
3. Eligible individuals were to be randomized to either of the eplerenone group or diuretic group with 1:1 proportion. To ensure allocation balance on baseline covariates (namely age, sex, and blood pressure), Pocock–Simon Minimization Algorithm^6^ was employed in randomization.

- Next, analyses of primary endpoints, secondary endpoints and safety were performed in the following analysis sets.
- Full Analysis Set (FAS): The FAS was defined as all randomized patients. The primary and secondary analyses were performed according to the randomized treatment.^[6]^Per Protocol Set (PPS): subset of the patients in the full analysis set who completed a certain pre-specified minimal exposure to the treatment regimen had some minimum number of measurements of the primary variable (s) had no major protocol violations
- Safety Analysis Set: The Safety Analysis Set was defined as all patients who received at least one dose of study drug on their actual treatment received.

Changes of primary endpoints were compared between the two groups using Analysis of Covariance. Difference between two groups were regarded as statistically significant if *P* < .05. Time-trends of endpoints during entire study period will be examined by employing mixed-effects models where baseline measurement of SBP, DBP, and heart rate levels as well as age and sex were used as covariates. All data analyses were carried out based on the intention-to-treat approach.

Data were independently and strictly monitored and analyzed by the data center (Academic Research Organization, Kurume University).

## 4 Results

### 4.1 Characteristics of parameters at baseline

A total of 204 patients were assigned by the dynamic allocation methods^[6]^ to the eplerenone (*n* = 102) and trichlormethiazide therapies (*n* = 102) (Table 1). Sixteen subjects were dropped out in the eplerenone group and 23 subjects were also dropped out in the trichlormethiazide group. Eventually, 165 patients (86 and 79 patients for each group) were enrolled in the present study. The mean age of participants was 64.8 years in eplerenone group (Table 2) and 65.1 years in trichlormethiazide group (Table 3). At baseline, BP and heart rate levels were comparable between the two groups. The 2 groups had similar characteristics, with an exception of HOMA-IR, adiponectin, and RLP-c levels (Tables 2 and 3).

**Table 1:**
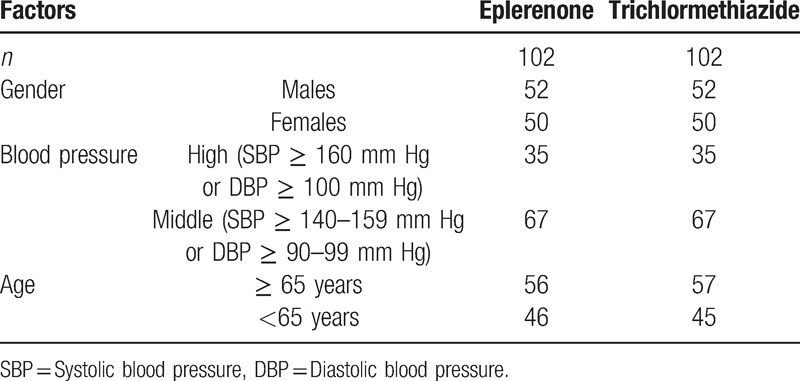
Allocation balance on baseline covariates.

**Table 2:**
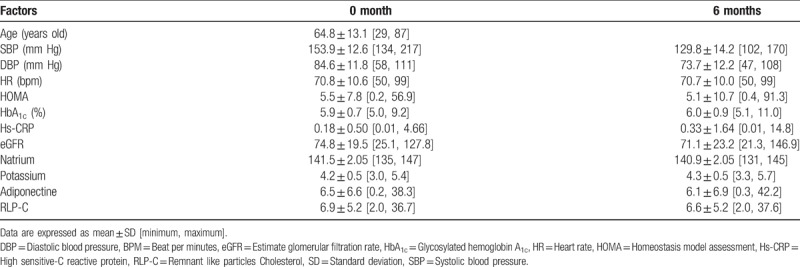
Characteristics of parameters at baseline and at 6 months in eplerenone group.

**Table 3:**
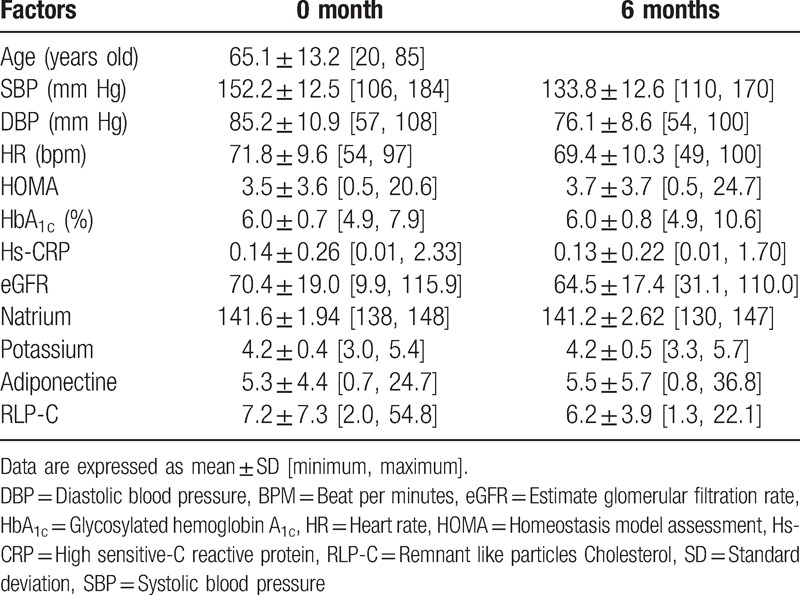
Characteristics of parameters at baseline and at 6 months in trichlormethiazide group.

### 4.2 Comparison between eplerenone and trichlormethiazide groups at baseline and at 6 months

**Figure 1:**
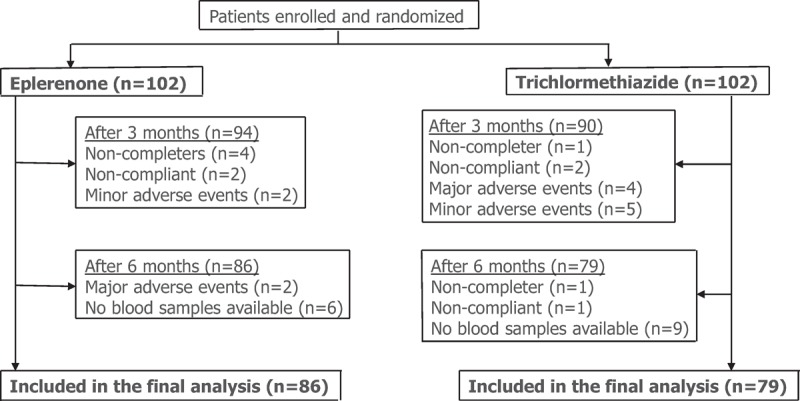
Recruitment of patients.

**Figure 2:**
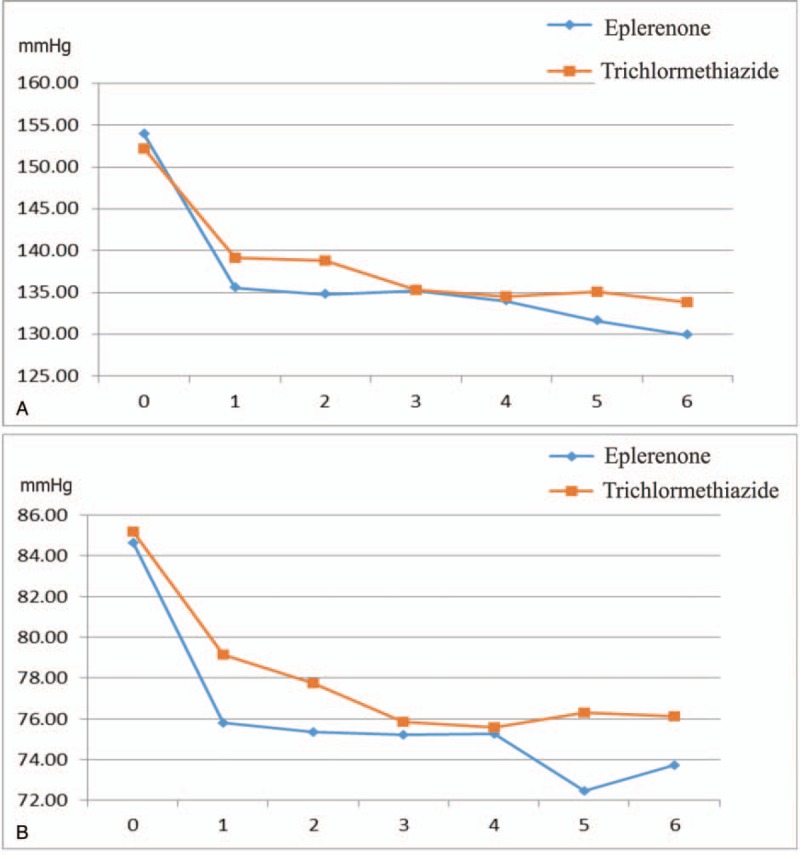
Change of systolic (A) and diastolic (B) blood pressures.

**Figure 3:**
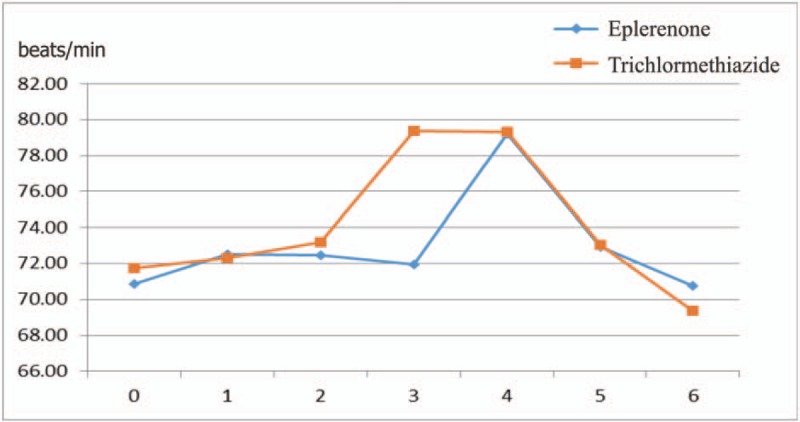
Change of heart rate.

**Figure 4:**
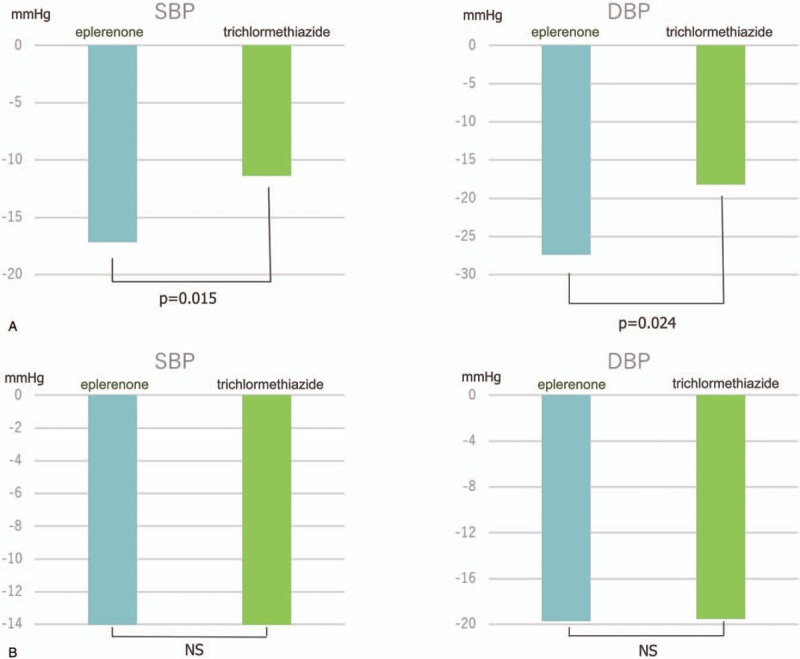
BP reduction between eplerenone and trichlormethiazide aged over 65 years (A) and under 65 years (B).

Figure 1 shows the recruitment and retention of patients. At 6 months, laboratory samples were available for 189 patients (92.6%; 96 in eplerenone therapy, 93 in trichlormethiazide therapy). There were 4 noncompleters, 2 complaints, and 4 minor and major adverse events in the eplerenone group, and 2 noncompleters, 3 complaints, and 9 minor and major adverse events in the trichlormethiazide group. Eventually, 86 patients in the eplerenone and 79 patients in the trichlormethiazide groups were included in the final analysis. Finally, eplerenone and trichlormethiazide were prescribed by 50–100 mg eplerenone and 1–2 mg trichlormethiazide, respectively. During 6 months of follow-up period, mean SBP was significantly lowered from 153.9 ± 12.6 to 129.8 ± 14.2 mm Hg in the eplerenone (*P* < .001) and from 152.2 ± 12.5 to 133.8 ± 12.6 mm Hg in the trichlormethiazide (*P* < .001) (Fig. 2**A**). Mean DBP significantly reduced from 84.6 ± 11.8 to 73.7 ± 12.2 mm Hg in the eplerenone (*P *< .001) and from 85.2 ± 10.9 to 76.1 ± 8.6 mm Hg (*P *< .001) in the trichlormethiazide (Fig. 2B). However, there were no significant changes in mean heart rate (from 70.8 ± 10.6 to 70.7 ± 10.0 mm Hg in the eplerenone and from 71.8 ± 9.6 to 69.4 ± 10.3 mm Hg in the trichlormethiazide (Fig. 3). The analysis of covariance adjusted for age, sex, and BMI indicated that SBP levels in the eplerenone were significantly lower than the trichlormethiazide (Table 4, *P* = .034). Potassium levels in eplerenone therapy were significantly higher (*P* = .002) than trichlormethiazide therapy. The SBP and DBP lowered levels between eplerenone and trichlormethiazide stratified by age groups (≥65 and <65 years) were demonstrated in Fig. 4A and B. Comparing patients aged under 65 years, SBP (*P* = .015) and DBP (*P* = .024) levels in patients aged over 65 years of eplerenone therapy were significantly lower than trichlormethiazide therapy. However, glucose metabolism does not show a significant difference between the two groups.

**Table 4:**
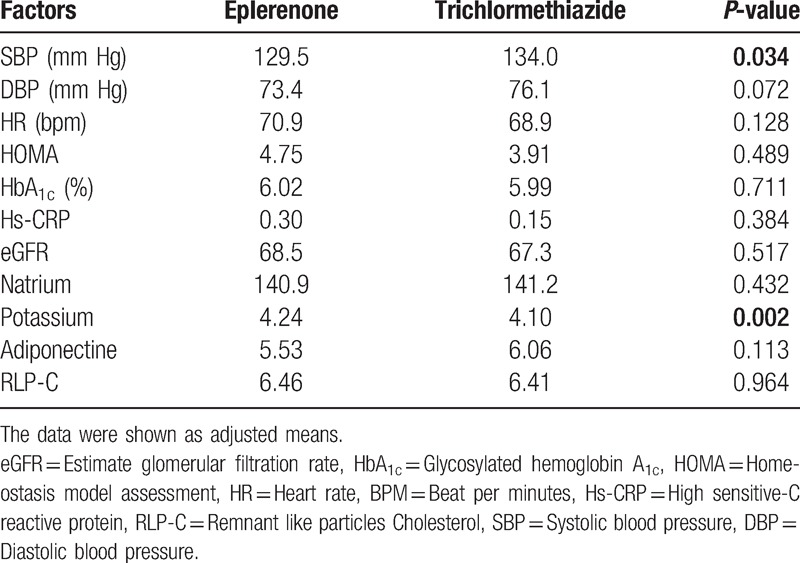
Comparison between eplerenone and trichlormethiazide by the analysis of covariance adjusted for age, sex, and BMI.

## 5 Discussion

This study provides the first evidence for the efficacy and safety of eplerenone therapy even in a relatively high age group, which demonstrated that 50–100 mg eplerenone therapy significantly lowered SBP in patients with hypertension and with overweight or obesity, especially in patients aged over 65 years, compared with 1–2 mg of trichlormethiazide.

### 5.1 Efficacy and safety of eplerenone therapy

Although we compared between 50 and 100 mg of eplerenone and 1–2 mg of trichlormethiazide in this study, Krum et al^[7]^ reported that the eplerenone may be useful add-on therapy in hypertensive patients. Eplerenone doses of 50–400 mg once daily are well tolerated and effective in reducing BP in patients with mild-to-moderate hypertension during a 24-hour period.^[8]^ On the other hand, efficacy of eplerenone versus enalapril as monotherapy in systemic hypertension was evaluated by Williams et al^[9]^ which demonstrated that eplerenone was as effective as enalapril for monotherapy in patients with stage 1 or 2 hypertension. Efficacy and safety of eplerenone therapy were also evaluated in Japanese patients with hypertension.^[10–12]^ The report from Saruta et al^[10]^ was a randomized, double-blind, placebo-controlled, parallel-group, dose-ranging study in 193 patients. Eplerenone has been shown to be an efficacious and well-tolerated agent and should be considered as one of the options for the management of patients with essential hypertension. The manuscript by Sato et al^[11]^ reported that the add-on therapy in Japanese patients with essential hypertension had an extremely beneficial antihypertensive effect, when eplerenone was co-administered with an ACE-I or long-acting CCB. Tsutsui et al^[12]^ also reported eplerenone was well tolerated in Japanese patients with heart failure with reduced ejection fraction.

As for safety, some studies are focusing on hyperkalemia.^[13–15]^ Sato et al^[13]^ reported a randomized controlled study of finerenone versus eplerenone in Japanese patients with worsening chronic heart failure and diabetes and/or chronic kidney disease. They found that mean changes in serum potassium levels were similar between finerenone and eplerenone. Khosla et al^[14]^ suggested that caution should be paid when using eplerenone for BP control in patients with advanced stage 3 nephropathy with a serum potassium of 4.5 mEq/l. Eschalier et al^[15]^ also recommended that eplerenone, including in patients with eGFR > 30 ml/min/1.73 m^2^ and potassium <5.0 mmol/l, was both efficacious and safe when carefully monitored in patients with chronic heart failure and reduced ejection fraction. Regarding the efficacy and safety of eplerenone in the management of mild-to-moderate hypertension, systematic review and meta-analysis by Pelliccia et al^[16]^ cautioned the increased potassium levels; however, average incidence of hyperkalemia (i.e. >5.5 mmol/l) in the all previous reports was below 2%. Only 0.1 mmol/l as average potassium increase in eplerenone group was found in our study. When we compared between eplerenone and trichlormethiazide by the analysis of covariance adjusted for age, sex, and BMI, a significant potassium increase (*P* = .002) was found in eplerenone group.

### 5.2 Eplerenone and glucose metabolism

Our previous study in a general population demonstrated that high levels of plasma aldosterone predicted the development of insulin resistance after 10 years in subjects without insulin resistance at baseline.^[4]^ Therefore, this study examined whether eplerenone, a selective aldosterone blocker, can improve the glucose metabolism such as insulin resistance. There have been some reports concerning the beneficial effect of eplerenone on glucose metabolism or metabolic syndrome. Bender et al^[17]^ suggested in their review that aldosterone and mineralocorticoid receptor (MR) signaling represent an ideal candidate pathway linking early promoters of diabetes, especially obesity, to vascular insulin resistance. McMurray et al^[18]^ also suggested reassuring evidence of the neutral effect of eplerenone on insulin action in hypertensive, nondiabetic patients. However, negative data have been also reported, in which Hwang et al^[19]^ did not support a contributing role for MR in endothelial dysfunction and insulin resistance in older adults with metabolic syndrome. Thus, as Tirosh et al^[20]^ also suggested that the evidence of MR blockade in metabolic syndrome are still scant, although several clinical trials have demonstrated the beneficial effects of MR antagonists on heart failure, hypertension, and diabetic nephropathy.^[21–23]^ On the other hand, Japanese investigators have reviewed the effects of eplerenone on salt-sensitive hypertension and chronic kidney disease and metabolic syndrome.^[24,25]^ The present study also did not show a beneficial effect on insulin resistance or metabolic syndrome in the eplerenone group, which might be caused by the insufficient period (6 months) to improve the metabolic-related factors.

### 5.3 Study strengths

The strengths of the current study are the use of PROBE design, multicenter trial, in which data were independently and strictly monitored and analyzed by statistics experts. Moreover, our data significantly contribute to scientific progress in a balanced perspective to the proposed important pathophysiological role of MR activation in insulin resistance and in metabolic syndrome although we could not indicate the beneficial effect.

### 5.4 Study limitations

First, we have studied a small number of Japanese hypertensives with obesity. Of the enrolled 204 patients, 39 subjects were dropped out. Second, although the readers may be interested in the 24 h, night, and day BP changes, the data of SBP and DBP were obtained only by office measurement. Third, since we did not perform echocardiology to the enrolled patients, eplerenone may have a different efficacy in patients affected by heart failure. Finally, our results might have been influenced by adherence of eplerenone and trichlormethiazide of the patients.

## 6 Conclusions

Our data demonstrated, for the first time using PROBE, that the eplerenone therapy was more effective for hypertension with overweight or obesity than the trichlormethiazide therapy, especially in elderly patients.

## Acknowledgments

We thank Drs. Bekki H (Bekki Clinic), Uemura S, Ohga M, Matsumoto M (Uemura Hospital), Onitsuka I (Tanushimaru Central Hospital), Shihara M, Kaneyuki M, (Ohmuta City Hospital), Jinnouchi J, Jinnouchi Y, (Sasaguri Hospital), Tamai O (Kasuya Minami Hospital), Kusaba K, Shimada T (Yame Public Hospital), Sugi K, Katsuki S (Sugi Hospital), Wada Y, Eguchi H (Wada Clinic), Tahara N (Kumashiro Hospital), Kumagai E, Okina N (Social Insurance Tagawa Hospital), Ohuchida M, Umei T (Chikugo City Hospital), Kai H (Yokokura Hospital), Yokota T, Yoshida T (Yokota Hospital), Miyamoto T (Chikugogawa-Onsen Hospital), Kanaya S, Yokoyama S (Sasebo Kyosai Hospital), Nohara Y (Yanagawa Hospital), Miyake Y (Akama Hospital), Kuwano K (Kuwano Clinic), Nagata T, Takeshita N (Higashi Saga Hospital), Morita H (Kurume Sogo Hospital), Yamamoto K (Yamamoto Clinic), Nakao K, Ohbu K (Ko-radai Rehabilitation Hospital), Nakamura T (National Kyusyu Medical Center), Tokuda K (Tokuda Clinic), Maki S (National Omuta Hospital), Ishizaki T (Ishizaki Clinic), Hashimoto Y (Miki Hospital), Nohara M (Nohara Clinic). We also thank Mrs. Makiko Tokuyasu for her secretarial assistance.

## Author contributions

**Data curation:** Eita Kumagai.

**Formal analysis:** Tatsuyuki Kakuma, Machiko Kawaguchi.

**Investigation:** Hisashi Adachi.

**Supervision:** Yoshihiro Fukumoto.

**Writing – original draft:** Hisashi Adachi.

## Supplementary Material

Supplemental Digital Content
