# Supplementary material for: Effects of eplerenone on blood pressure and glucose metabolism in Japanese hypertensives with overweight or obesity
Source: Medicine (Baltimore). 2019 Apr 12;98(15):e14994. doi: 10.1097/MD.0000000000014994 (PMC6485869; doi:10.1097/MD.0000000000014994)
Supplement: Supplemental Digital Content [file medi-98-e14994-s001.doc]

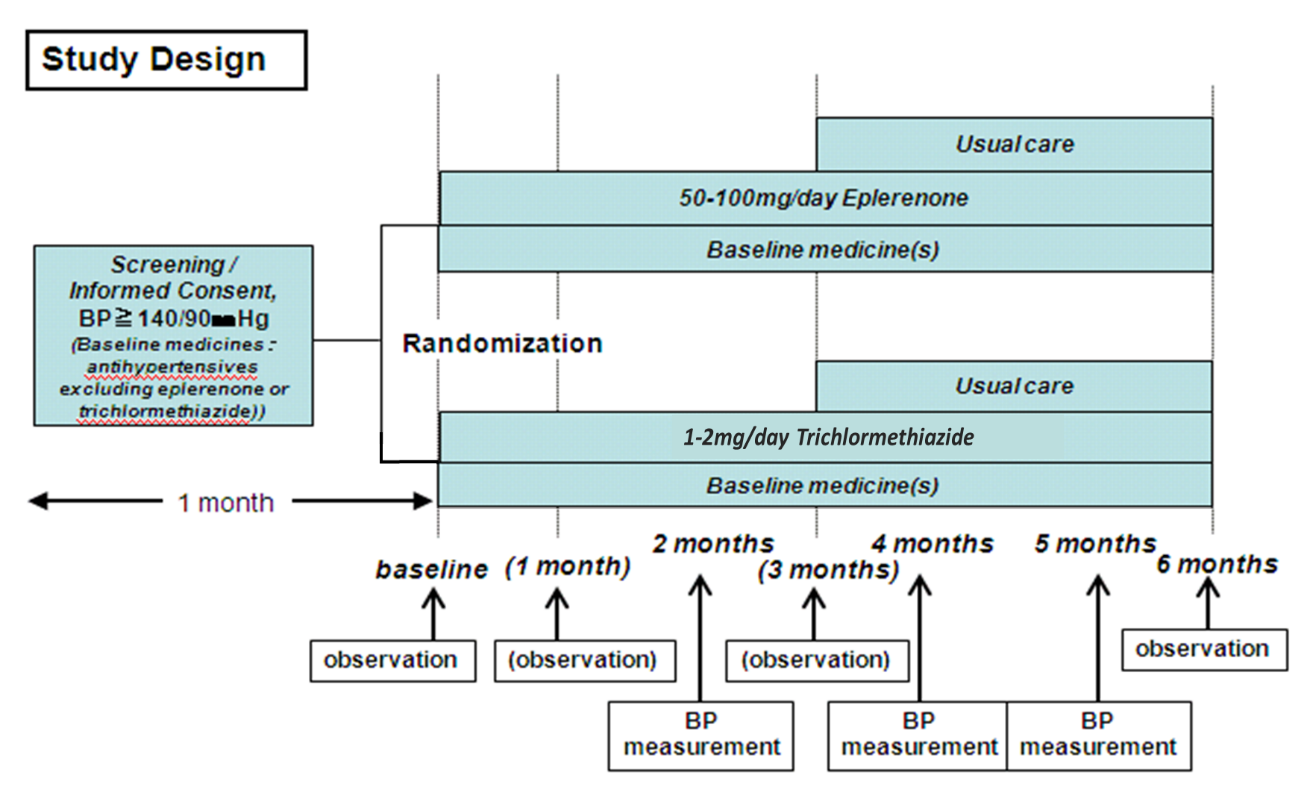


Supple 1

| Items | Screening | Trial period | | | | | | |
| --- | --- | --- | --- | --- | --- | --- | --- | --- |
| Period | -4 wks  (-1 mo) | 0  wk | 4 wk  (1 mo) | 8wk  (2mo) | 12 wk  (3 mo) | 16wk  (4mo) | 20wk  (5mo) | 24wk  (6mo) |
| Office visit | ○ | ○ | △ |  | △ |  |  | ○ |
| Screening items | ○ |  |  |  |  |  |  | ○ |
| Informed Consent | ○ |  |  |  |  |  |  |  |
| Randomization |  | ○ |  |  |  |  |  |  |
| Office BP, HR | ○ | ○ | ○ | ○ | ○ | ○ | ○ | ○ |
| Blood test | ○ | ● |  |  |  |  |  | ○ |
| Serum potassium | ○ | ○ | △ |  | △ |  |  | ○ |
| Patient background | ○ | ● |  |  |  |  |  |  |
| Physical findings (incl. abdominal circumference) | ○ | ● | △ |  | △ |  |  | ○ |
| Concomitant meds & therapies | ○ | ● |  |  |  |  |  | ○ |
| Renal function |  | ○ |  |  |  |  |  | ○ |
| 12 Lead ECG |  | △ |  |  |  |  |  | △ |
| Primary endpoints |  | ○ |  |  | △ |  |  | ○ |
| Secondary endpoints |  | ○ |  |  | △ |  |  | ○ |
| Study drug compliance check |  |  | △ |  | △ |  |  | ○ |
| adverse events |  |  | △ |  | △ |  |  | ○ |

●: Not necessary to measure if measured at screening.

○: Measure within +/- 2 weeks of the targeted period

△：Measure within +/- 2 weeks of the targeted period if possible

BP measurement should be done monthly
